# Chromosome-level genome assembly and annotation of a novel potato common scab pathogen

**DOI:** 10.3389/fpls.2026.1749870

**Published:** 2026-03-13

**Authors:** Lingling Wei, Jin Pu, Hui Du, Rongyan Wang, Xiaowenxuan Gao, Qiangbiao Zhao, Tianjie Wang, Jianli Gao, Decai Yu, Guangtao Zhu, Jing Liu

**Affiliations:** 1 Yunnan Key Laboratory of Potato Biology, School of Life Sciences, Yunnan Normal University, Southwest United Graduate School, Kunming, Yunnan, China; 2 School of Pharmaceutical Sciences, School of Pharmacy and Academy of Chinese Medical Science, Zhejiang Chinese Medical University, Hangzhou, Zhejiang, China; 3 College of Plant Protection, Wenshan Academy of Agricultural Sciences, Wenshan, Yunnan, China; 4 College of Plant Protection, Yunnan Agricultural University, Kunming, Yunnan, China

**Keywords:** comparative genomics, gapless genome assembly, pathogenicity, potato common scab, *Streptomyces lincolnensis*, sister species, virulence genes

## Abstract

**Introduction:**

Potato common scab, caused by pathogenic *Streptomyces* species, severely impairs tuber quality and restricts potato industry development. *Streptomyces lincolnensis* is traditionally known for lincomycin biosynthesis, with no prior association with plant pathogenicity.

**Methods:**

In this study, 31 actinomycete strains were isolated from scab-infected potato tubers, and strain D6 was identified as a highly virulent pathogen. Chromosome-level complete genome sequencing of strain D6 was performed, followed by phylogenetic and phylogenomic analyses. Average nucleotide identity (ANI) and digital DNA-DNA hybridization (dDDH) were used to determine its taxonomic status. Comparative genomic analysis was conducted between strain D6, pathogenic *Streptomyces scabiei*, and non-pathogenic *S. lincolnensis* to identify virulence-related genes.

**Results:**

Phylogenomic analyses confirmed that strain D6 belongs to the *S. lincolnensis* clade. ANI and dDDH values between strain D6 and strain *S. lincolnensis* NRRL 2936 were 87.2% and 28.9%, respectively, indicating that strain D6 represents a novel sister species of *S. lincolnensis*. Comparative genomics further revealed a core set of 74 virulence-associated genes shared by strain D6 and the typical pathogen *S. scabiei* LBUM848, but absent in the non-pathogenic *S. lincolnensis* NRRL 2936. Four key candidate virulence genes were screened from this core set: *PROKKA_06934* (encoding a class III pyridoxal-phosphate-dependent aminotransferase), *PROKKA_02771* (a *TxtC* homolog, encoding a cytochrome P450 enzyme), *PROKKA_05140* (a phage-derived gene), and *PROKKA_08104* (encoding a glycoside hydrolase family 32 protein).

**Discussion:**

These findings provides both experimental and genomic evidence supporting that strain D6, a novel sister species of *S. lincolnensis*, is a causal agent of potato common scab. This study expands the diversity of plant-pathogenic *Streptomyces* and offers genomic insights into the evolution of virulence in pathogenic *Streptomyces* species.

## 1 Introduction

The potato (*Solanum tuberosum* L.) is a globally important staple crop rich in carbohydrates, dietary fiber, vitamins, and essential minerals, ranking fourth among food crops worldwide after rice, wheat, and maize (Zhang et al., 2023). However, its production is threatened by potato common scab, a widespread soil-borne bacterial disease first described by Thaxter in 1891 (Thaxter, 1891) and subsequently reported in most major potato-producing regions worldwide (Lambert and Loria, 1989; Dees et al., 2013; Wanner, 2006). It is currently regarded as one of the most economically important potato diseases globally. Typical symptoms include brown, rough, and cracked lesions on the tuber surface, which significantly reduce tuber quality and market value, thereby posing a persistent threat to sustainable potato production (Park et al., 2003; Al-Mughrabi et al., 2015).

The causal agents of potato common scab are pathogenic species belonging to the genus *Streptomyces*. Among the 767 species and subspecies of *Streptomyces* identified to date, the majority are well recognized for producing antibiotics and other secondary metabolites (Kaneko et al., 2024). However, several *Streptomyces* species have evolved pathogenic capabilities, and pathogenicity varies substantially across different strains (Ismail et al., 2020; Ghuffar et al., 2025). The most frequently reported potato common scab pathogens include *Streptomyces scabies*, *Streptomyces acidiscabies*, and *Streptomyces turgidiscabies* (Lapaz et al., 2012; Nishi et al., 2015; Tomihama et al., 2016). To date, more than ten *Streptomyces* species have been associated with potato common scab, but the pathogenic mechanism is highly conserved across these pathogenic species. Most pathogenic *Streptomyces* strains are capable of producing thaxtomin, a phytotoxic secondary metabolite and a well-characterized virulence factor for potato common scab (Tomihama et al., 2016; Francis et al., 2015). The genes responsible for thaxtomin biosynthesis (e.g., *txtAB*, *txtC*, *nos*, *txtR*, *tomA*, and *nec1*) are typically located on a pathogenicity island (PAI) (Scheible et al., 2003; Healy et al., 2002; Liu et al., 2023). Importantly, PAIs are mobile genetic elements, and their horizontal transfer between pathogenic and non-pathogenic *Streptomyces* strains reshapes virulence gene repertoires, enhances genomic diversity, and contributes to the emergence of novel pathogenic lineages (Kers et al., 2005; Zhang and Loria, 2017; Weisberg et al., 2023). Recent work has identified toxin-like secondary metabolites such as coronafacic acid like compounds and multiple auxiliary virulence factors that contribute to scab pathogenesis (Francis et al., 2023; Liu et al., 2023, 2021). These auxiliary determinants include regulatory proteins modulating thaxtomin biosynthesis, antibacterial agents such as bottromycins and concanamycins, siderophores, plant cell wall degrading enzymes, and elicitor transporters that mediate host colonization and tissue penetration. Notably, stain *Streptomyces* sp. 11-1–2 lacks the ability to produce thaxtomin A but can synthesize nigericin and geldanamycin, which are also phytotoxic to plants (Díaz-Cruz et al., 2022).

*Streptomyces lincolnensis*, a notable actinomycete within the *Streptomyces* genus, has attracted substantial research attention due to its unique capacity to synthesize lincomycin, a clinically important lincosamide antibiotic. Current research has primarily focused on elucidating key regulatory factors in the lincomycin biosynthetic pathway. For instance, deletion of the pleiotropic transcriptional regulator AdpA_lin_ disrupts lincomycin biosynthesis and impairs morphological differentiation (Kang et al., 2019). Additionally, the TetR-type regulator SLCG_2919 has been identified as a direct repressor of lincomycin production, whereas AtrA_lin_ functions as a positive regulator of this process (Xu et al., 2018). Notably, no previous studies have reported an association between *S. lincolnensis* or its closely related species and potato common scab. A study published in 2017 described *S. lincolnensis* but did not investigate its pathogenicity, nor did it include the genomic sequence in the article, although the genome data were subsequently deposited in the NCBI database (Hou et al., 2017). In this study, we provide the first experimental evidence demonstrating the pathogenicity of a novel sister species of *S. lincolnensis* (strain D6) toward potato common scab.

High-quality genome sequencing and assembly of pathogenic microorganisms are crucial for understanding their virulence mechanisms, epidemiology, and evolutionary trajectories. The rapid advancement of sequencing technologies, particularly the emergence of third-generation sequencing platforms such as PacBio HiFi (High Fidelity), has revolutionized genomic studies of pathogens. Characterized by long read lengths and high base-level accuracy, HiFi sequencing data substantially enhance the continuity and completeness of genome assemblies, laying a robust foundation for downstream in-depth analyses. Highly continuous genomes facilitate the comprehensive identification of key genomic elements, including PAIs, virulence factor genes, antibiotic resistance genes, and mobile genetic elements, which are essential for deciphering pathogenicity and antimicrobial resistance. For instance, in studies focusing on pathogens such as *Vibrio parahaemolyticus* and *Staphylococcus aureus*, PacBio HiFi data have markedly enhanced assembly contiguity, enabling more complete characterization of virulence determinants and antibiotic resistance genes (Lee et al., 2024; Deng et al., 2019). Similarly, genomic investigations of *Salmonella gallinarum* have provided insights into its repertoire of antibiotic resistance genes and mobile genetic elements, while HiFi-based assembly of *Pseudomonas syringae* genomes has uncovered genes associated with pathogenicity and host specificity (McCann, 2016; Jia et al., 2025).

In this study, 31 strains were isolated from potato common scab infected tubers, with strain D6 selected for subsequent analyses for its highest inhibitory activity against radish seedlings. Phylogenetic analysis of the 16S rRNA gene indicated that strain D6 and *S. lincolnensis* formed a single clade. High-quality genome sequencing and comparative genomic analysis have emerged as powerful tools for identifying bacterial virulence determinants, yet the lack of chromosome-level genome assemblies and comparative analyses between pathogenic and non-pathogenic strains of the *S. lincolnensis* clade have hindered our understanding of pathogenicity evolution. To address these problems, we generated a gapless, high-quality genome assembly of D6 using HiFi long-read sequencing. Comparative genomics between pathogenic strain D6, non-pathogenic *S. lincolnensis* NRRL2936, and pathogenic *S. scabiei* LBUM848 were then conducted, aiming to identify conserved virulence genes, genomic islands (GI) and key factors driving pathogenicity. Four candidate virulence factors were further characterized. Collectively, this study presents the first integrated genomic and experimental evidence supporting that strain D6, a novel sister species of *S. lincolnensis*, is a causative agent of potato common scab, providing crucial insights into the molecular mechanisms of virulence evolution in plant-pathogenic *Streptomyces* species.

## 2 Materials and methods

### 2.1 Growth conditions

RH hybrid and RH inbred potato plants were initially cultivated in non-sterile substrate soil in the greenhouse. The first phase aimed to isolate potential pathogenic bacteria from naturally infected tubers. Subsequently, to fulfill Koch’s postulates with the isolated strains, a separate experiment was conducted using sterilized substrate soil. This sterilization step was critical to eliminate interference from other soil-borne pathogens, and the plants were inoculated via root irrigation with the purified strains.

### 2.2 Isolation and purification of the potato common scab pathogens

Diseased potato tubers with typical common scab lesions were collected, rinsed thoroughly under running water, and air-dried at room temperature. Under aseptic conditions, tissue fragments were extracted from the lesion margins and surface-sterilized via sequential immersion in 75% ethanol and 1% NaClO. The sterilized fragments were rinsed 3 times with sterile distilled water to remove residual disinfectants, blotted dry with sterile filter paper, and placed on oatmeal agar (OMA) medium, followed by incubation at 28 °C in darkness. After 5–7 days, emerging *Streptomyces* colonies were purified via three consecutive rounds of subculturing on fresh OMA medium to obtain pure strains.

### 2.3 Pathogenicity assays

#### 2.3.1 Potato tuber slice assay

Healthy potato tubers were surface-sterilized and cut into uniform slices (1.5 cm in diameter and 2–3 mm in thickness). The slices were placed on moist sterile filter paper in Petri dishes to maintain humidity. Mycelial plugs (5 mm in diameter) were obtained from 10-day-old pure cultures of the test strains and aseptically placed onto the center of each potato slice. For the control group, sterile OMA plugs were used in place of mycelial plugs. All Petri dishes were incubated at 28 °C in darkness, and symptom development was assessed after 3–5 days. The browning and necrotic areas on potato slices were quantified using ImageJ (v1.49) software (National Institutes of Health, USA). To prevent assessment bias, the image analysis was conducted in a blinded manner. All slices were photographed under standardized lighting conditions with a scale bar included. During analysis, the diameter of potato slice was first used as a reference for calibrating the image scale, after which the proportion of browning area of each slice was measured and quantified.

#### 2.3.2 Radish seedling assay

Bacterial suspensions were prepared by inoculating single pure colonies into Gauze’s No. 1 liquid medium, followed by incubation at 28 °C with shaking at 220 rpm for 2 days. Radish seeds were surface-sterilized with 2% NaClO for 2 minutes, then rinsed 3 times with sterile water. The sterilized seeds were placed on moistened sterile triple-layered filter paper in Petri dishes to promote germination. After 1–2 days, uniformly germinated seeds were transferred to tubes containing 1.8% water agar and inoculated with 200 µL of the bacterial suspension (1×10^7^ CFU/mL); control groups received only sterile medium. After cultivating the seedlings under a 16-hour photoperiod for 6–10 days, the seedling length was measured, and the inhibition rate was calculated using the formula:

Inhibition rate (%) = (Control length - Treatment length)/Control length × 100%. Each treatment was conducted with 3 biological replicates.

### 2.4 Morphological characterization

The purified *Streptomyces* strains were inoculated onto OMA medium and incubated at 28 °C in darkness for 10 days. Subsequently, the color and morphology of the spores and sporulating structures were observed. A single colony was carefully transferred to a sterile glass slide using a dissecting needle and covered with a coverslip to allow the sporulation structures and aerial mycelia to adhere. The morphology of the spore chains and aerial mycelial branching patterns were then examined under a light microscope.

### 2.5 Re-isolation and verification via Koch’s postulates

The potato plants were cultivated in a controlled greenhouse environment with a growth substrate consisting of peat soil and perlite, under an average daily temperature range of 18 °C to 25 °C during the growth period. Pot inoculation was conducted using a root irrigation method. Virus-free plantlets derived from RH self-pollinated progeny tubers were acclimatized and transplanted into pots after 2 weeks. Bacterial spores were harvested from D6 cultured on OMA medium for approximately 10 days by rinsing with 100 mL of sterile water. The spore suspension was adjusted to a concentration of 1×10^7^ CFU/mL. Each pot was inoculated with 150 mL of the suspension via root irrigation, while control plants received an equal volume of sterile water. The experiment included 6 replicates per treatment, and all plants were maintained in the same greenhouse. A short-term drought stress was applied 4–6 weeks post-inoculation. At maturity, tubers were harvested and assessed for disease incidence.

To fulfill Koch’s postulates, diseased tubers with severe symptoms were selected. The pathogen was re-isolated from the lesions using the same method described previously. The re-isolated strain was then identified and compared morphologically with the original inoculum.

### 2.6 High-fidelity sequencing, genome assembly, quality assessment and annotation

Following purification, strain D6 was submitted to Annoroad Gene Technology (Beijing) Co., Ltd. for PacBio HiFi whole-genome sequencing. Genome assembly was performed using Flye (v2.7.1-b1590) with HiFi reads as input, followed by polishing with Pilon (v1.24) to correct potential errors. Genome statistics were calculated using Seqkit (v2.4.0), and completeness was assessed via BUSCO (v5.5.2) against the *Streptomycetales_odb10* dataset. For genome annotation, Prokka (v1.14.6) was used to integrate gene prediction, rRNA/tRNA identification, and protein function annotation. Functional annotation was carried out using multiple databases, including NCBI NR, EggNOG, COG, PFAM, KEGG, GO, and SwissProt. Additionally, specialized databases—PHI-base (v4.15), Virulence Factor Database (VFDB, core and complete datasets), Comprehensive Antibiotic Resistance Database (CARD, v3.2.8), Carbohydrate-Active Enzymes Database (CAZy), and a prophage virus database—were employed to identify virulence-related genes, antibiotic resistance genes, and carbohydrate-active enzyme (CAZyme) genes. Annotation statistics were visualized using R (v4.3.0) and the ggplot2 package.

### 2.7 Phylogenetic, phylogenomic, average nucleotide identity and digital DNA-DNA hybridization analyses

Three complementary strategies were used to determine the phylogenetic position of strain D6 and related *Streptomyces* strains. First, phylogenetic trees based on 16S rRNA nucleotide sequences, gyrB and ropB amino acid sequences were constructed using MEGA11 with the maximum-likelihood method, and branch support was assessed with 1,000 bootstrap replicates. Second, a whole-genome phylogenomic analysis was performed based on 1,865 single-copy core genes (comprising 591,391 amino acid sites) identified by OrthoFinder (v2.5.4). Individual gene families were aligned using MAFFT (v7.520), and a maximum-likelihood tree was constructed from the concatenated alignment using IQ-TREE (v3.0.1) under the LG+I+R6 model, with branch support evaluated using 1,000 ultrafast bootstrap replicates. In addition, the genome-wide average nucleotide identity (ANI) between strain D6 and the type strain *S. lincolnensis* NRRL 2936 was calculated using the JSpeciesWS online service (https://jspecies.ribohost.com/jspeciesws/#analyse). Digital DNA-DNA hybridization (dDDH) values between strain D6 and *S. lincolnensis* NRRL 2936 were determined using the Genome-to-Genome Distance Calculator (GGDC) 3.0 web server (https://ggdc.dsmz.de) with the recommended formula 2.

### 2.8 Identification of virulence-associated genes, GIs, and prophages

Putative virulence-associated genes were identified by aligning coding sequences against specialized databases using BLASTp (v2.7.1+) with an E-value cutoff of 1e−5. The databases included the Prophage Virus database, PHI-base (v4.15), VFDB, CARD (v3.2.8), and CAZy. GIs were predicted using IslandViewer 4 (https://www.pathogenomics.sfu.ca/islandviewer/) with integrated IslandPath-DIMOB and SIGI-HMM algorithms. Prophage sequences were identified via PHASTEST (https://phastest.ca/).

### 2.9 Comparative genomic analysis and thaxtomin-related homolog identification

Whole genome synteny analysis among the 3 strains was performed using JCVI (v1.5.1). For comparative analysis, the nonpathogenic *S. lincolnensis* (NRRL2936) and the pathogenic reference strain *S. scabiei* (LBUM848) were retrieved from the NCBI database. Candidate thaxtomin biosynthetic genes were identified by BLASTp (v2.7.1+) comparison of D6 protein sequences against the LBUM848 thaxtomin gene cluster (*txtA*, *txtB*, *txtC*, *txtR*, n*os*, *tomA*, *nec1*) using an E-value threshold of 1e-10. Protein domain prediction was performed using InterProScan, and the results were visualized with IBS 2.0 software.

## 3 Results

### 3.1 Isolation and pathogenicity assessment of the pathogenic bacteria

**Figure 1:**
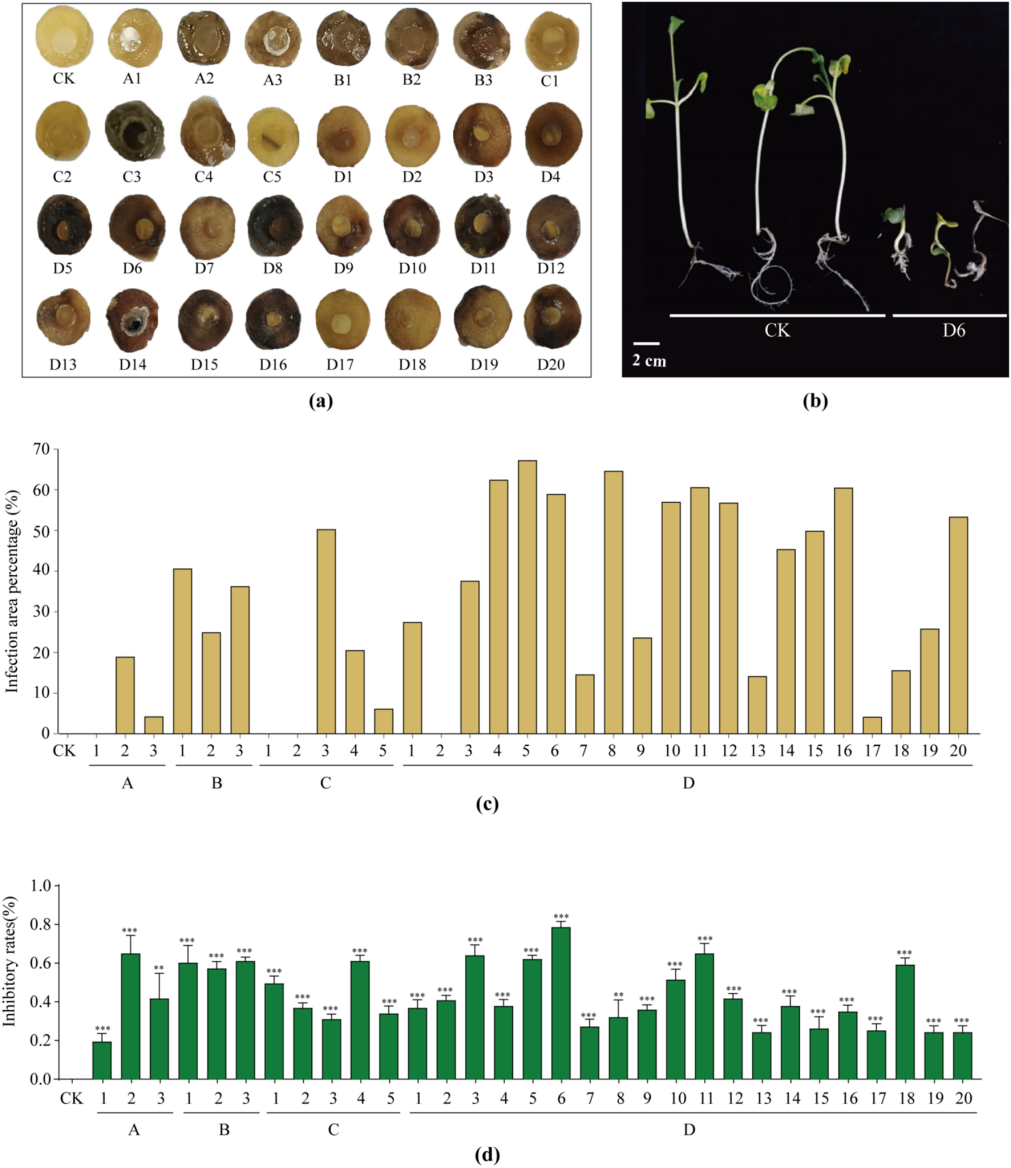
Pathogenicity identification of isolated strains. **(a)** Pathogenicity testing of 31 isolated strains on potato tuber slices. **(b)** Pathogenicity assay of strain D6 on radish seedlings. Scale bar, 2 cm. **(c)** The ratio of the diseased area to the total area in **(a)**. **(d)** Inhibition rate of different strains on radish seedlings. Statistical significance analysis was determined by two-tailed *t*-test with unequal variance. Thresholds for significance were defined as *P* < 0.01 (**) and *P* < 0.001 (***). Each treatment included 3 independent biological replicates.

A total of 31 actinomycetes strains were isolated and purified from diseased potato tubers (**Supplementary Table 1**). Pathogenicity tests were conducted on all 31 strains using the potato tuber slice assay, and the proportion of the diseased area quantified. After inoculation with different strains, potato tuber slices exhibited varying degrees of browning and necrotic lesions, which were typical symptoms of infection (**Figure 1a**). Among these strains, 20 caused the diseased area to exceed 20% (**Figure 1c**). Additionally, the radish seedling assay showed that all 31 strains exerted varying degrees of inhibitory effects on radish seedling growth, with inhibition rates ranging from 21% to 80% (**Figure 1d**). Among these, 9 strains exhibited growth inhibition rates exceeding 60% against radish seedlings, indicating strong inhibitory activity. Notably, strain D6 showed the highest inhibitory rate, reaching 80% (**Figures 1b, d**).

### 3.2 Phylogenetic analysis, morphological characterization and detection of the pathogenic genes

**Figure 2:**
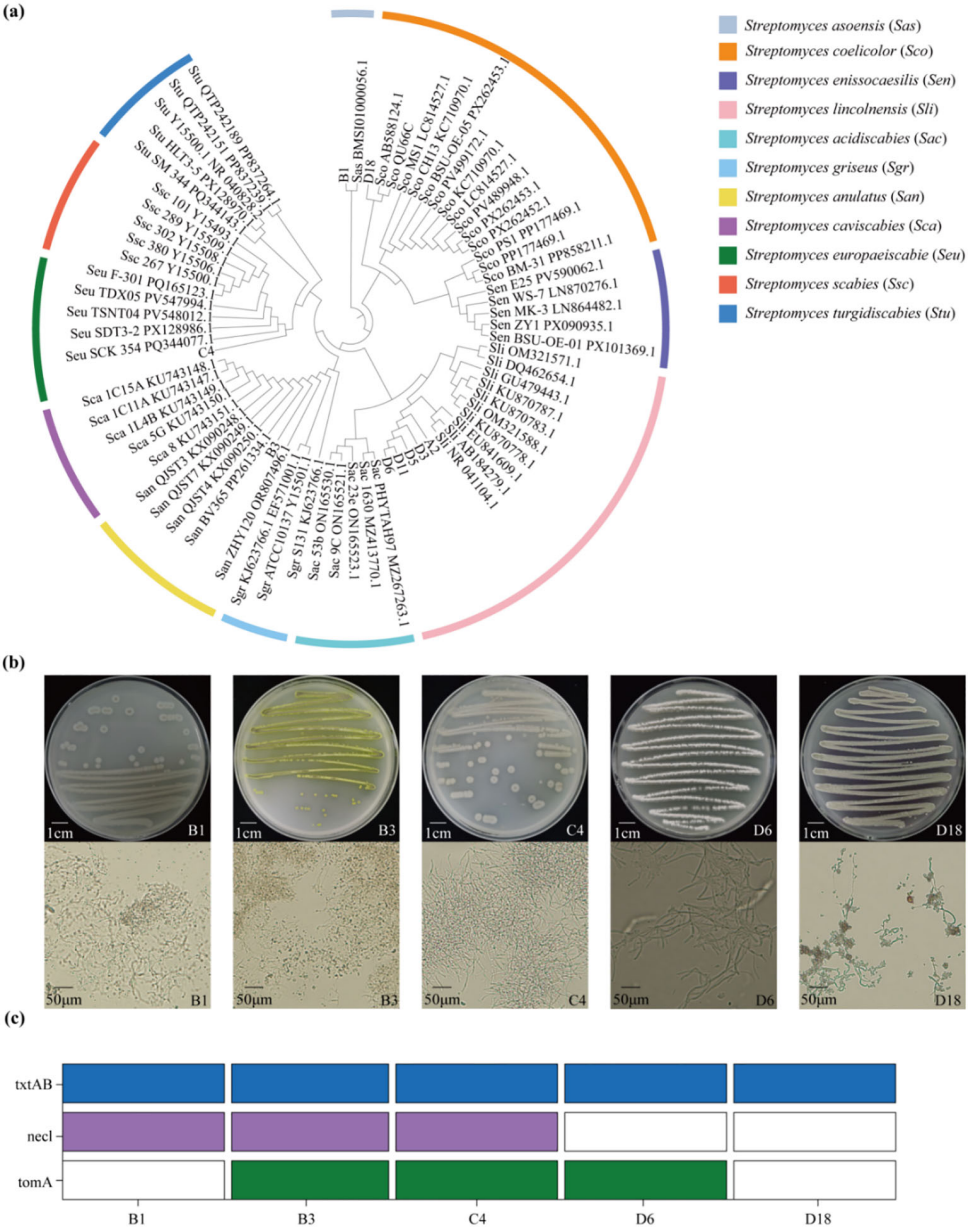
Phylogenetic analysis, morphological identification and pathogenic gene detection of the pathogenic strains. **(a)** Phylogenetic analysis of strain A2, B1, B3, C4, D3, D5, D6, D11, D18 and reported *Streptomyces* species. **(b)** The morphology of colonies, hyphae and spores of strain B1, B3, C4, D6, D18. The first row shows the colony morphology of the 5 strains. Scale bar, 1 cm. The second row shows the hyphae and spores morphology of the 5 strains under microscopy. Scale bar, 50 µm. **(c)** Schematic diagram of the genotype of pathogenic genes of the 5 strains in **(b)**. The presence or absence of the indicated pathogenic genes is represented by colored and uncolored boxes, respectively: *txtAB* (blue), *nec1* (purple), and *tomA* (green).

Among the tested strains, 9 exhibiting an inhibition rate of 60% or higher against radish seedling growth were selected for further study (**Figure 1d**). The 16S rRNA gene was amplified from these 9 strains using the universal primers 27F and 1492R, yielding a 1500 bp gene fragment (**Supplementary Figure 1a**). Following BLAST alignment of the sequencing results in GenBank, a phylogenetic tree was constructed using MEGA11 software. For phylogenetic tree construction, reference strains with >95% similarity to the query strains and previously reported *Streptomyces >scabies* strains were selected. Phylogenetic analysis revealed that strain B1 was closely related to *Streptomyces asoensis*, strain D18 to *Streptomyces coelicolor*, strain B3 to *Streptomyces anulatus*, and strain C4 to *Streptomyces europaeiscabiei* (**Figure 2a**). Notably, the 5 strains (A2, D3, D5, D11, D6) and *S. lincolnensis* formed a single, well supported clade, indicating a shared evolutionary lineage (**Figure 2a**). Furthermore, the compact branching pattern and high sequence similarity among these 5 strains suggest an exceptionally close genetic relationship, suggesting a recent common ancestor. Given this, the highly pathogenic strain D6 was selected as the representative strain for subsequent experiments.

Following pathogenicity verification and molecular biological identification (e.g., 16S rRNA sequence analysis), five representative *Streptomyces* strains (B1, B3, C4, D6, D18) were selected for in-depth morphological characterization and pathogenic gene detection. After cultivation on OMA medium, the strains formed colonies with distinct morphological features, including variations in shape, surface texture, and edge characteristics (**Figure 2b**). Specifically, strains B3 produced yellow spore masses, while the other 4 strains were white (strain D6) or grayish-white (strains B1, C4, and D18). Microscopic observation at 10×40 magnification revealed that all tested strains formed flat to flexible hyphae, while strain D18 was additionally observed to produce spiral spore chain (**Figure 2b**).

To further elucidate the pathogenic potential of these strains, PCR amplification was performed for pathogenicity-related genes associated with potato common scab. Previous studies have analyzed the PAI of pathogenic *Streptomyces* species and identified that the key pathogenic genes include *txtAB*, *nec1*, and *tomA* (Wanner, 2006). The *txtAB* was established as a determinant for pathogenicity, while the *nec1* and *tomA* were shown to influence the level of virulence. All five strains yielded positive amplification of the *txtAB* gene (encoding a key enzymes in thaxtomin biosynthesis), with a PCR product of approximately 385 bp (**Supplementary Figure 1b**). The *nec1* gene (potentially linked to host necrosis) was successfully amplified in strains B1, B3, and C4, producing a PCR product of approximately 700 bp (**Supplementary Figure 1c**). Meanwhile, the *tomA* gene (putatively involved in toxin transport) was amplified in strains B3, C4, and D6, resulting in an approximately 392 bp PCR product (**Supplementary Figure 1d**). Based on these results, the PAI genotypes for each strain were determined (**Figure 2c**): B1 (*txtAB^+^/nec1^+^/tomA^–^*), B3 (*txtAB^+^/nec1^+^/tomA^+^*), C4 (*txtAB^+^/nec1^+^/tomA^+^*), D6 (*txtAB^+^/nec1^–^/tomA^+^*), and D18 (*txtAB^+^/nec1^–^/tomA^–^*). These genotypic variations suggest that different strains may employ distinct pathogenic mechanisms.

### 3.3 Verification of pathogenicity via Koch’s postulates

**Figure 3:**
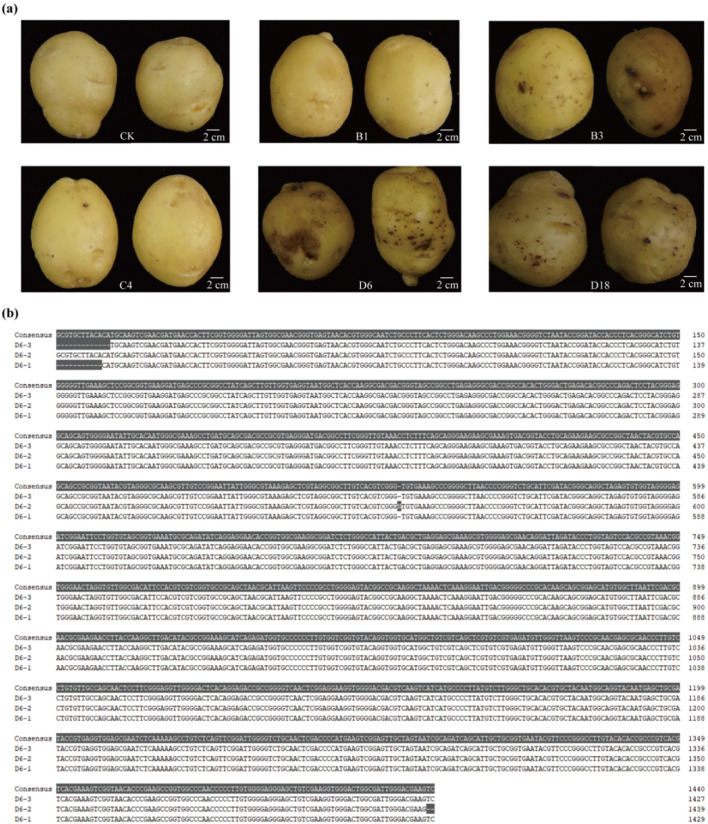
The strains were verified by Koch’s postulates. **(a)** Scab symptoms observed on potatoes inoculated with strains B3, D6, and D18. Scale bar, 2 cm. **(b)** Molecular identification and sequence alignment of the original strain D6 and re-isolated strains (D6–1 to D6-3).

To confirm the pathogenic potential of the selected strains, B1, B3, C4, D6, and D18 were inoculated onto pot-grown potato plants. Post-harvest inspection revealed that inoculation with strains B3, D6, and D18 resulted in distinct common scab symptoms on the tubers. Among these, strain D6 induced the most severe symptoms, whereas tubers inoculated with strains B1 and C4 remained asymptomatic (**Figure 3a**). These results confirm the pathogenicity of strains B3, D6, and D18, with D6 exhibiting particularly high virulence. In contrast, strains B1 and C4 were non-pathogenic under the tested experimental conditions. Since strain D6 has high virulence and belongs to the dominant gene cluster, and fully conforms to Koch’s postulates, we selected this strain for further study.

For the re-isolation step, the same initial isolation protocol was employed to recover three *Streptomyces* strains, designated D6-1, D6-2, and D6-3, directly from the scab lesions of tubers previously inoculated with strain D6. The conserved region of the 16S rRNA gene in strain D6 and 3 re-isolated strains was amplified using universal primers, and the resulting PCR products were subsequently sequenced. Multiple sequence alignment analysis demonstrated that these 3 re-isolated strains exhibited 99.9% sequence identity with the original inoculated strain D6 (**Figure 3b**; **Supplementary Table 2**). The successful re-isolation of D6 from symptomatic lesions, combined with its ability to induce disease upon re-inoculation, definitively fulfilled Koch’s postulates. Thus, strain D6 was confirmed as the causative agent of potato common scab.

### 3.4 Genomic characterization and phylogenetic identification of D6

To better identify the key virulence genes contributing to potato common scab, we performed *de novo* genome assembly of D6 using third-generation PacBio High-Fidelity (HiFi) long-read sequencing technology. A total of 4.5 Gb of high-quality data were generated, consisting of 227,696 reads with an average length of 19,868 bp. Given the estimated genome size of approximately 10 Mb, this corresponded to a sequencing depth of 452×. The resulting assembly represents the first gapless, chromosome-level genome map of D6, providing a robust foundation for elucidating its genomic architecture and pathogenic potential (**Supplementary Table 3**).

**Figure 4:**
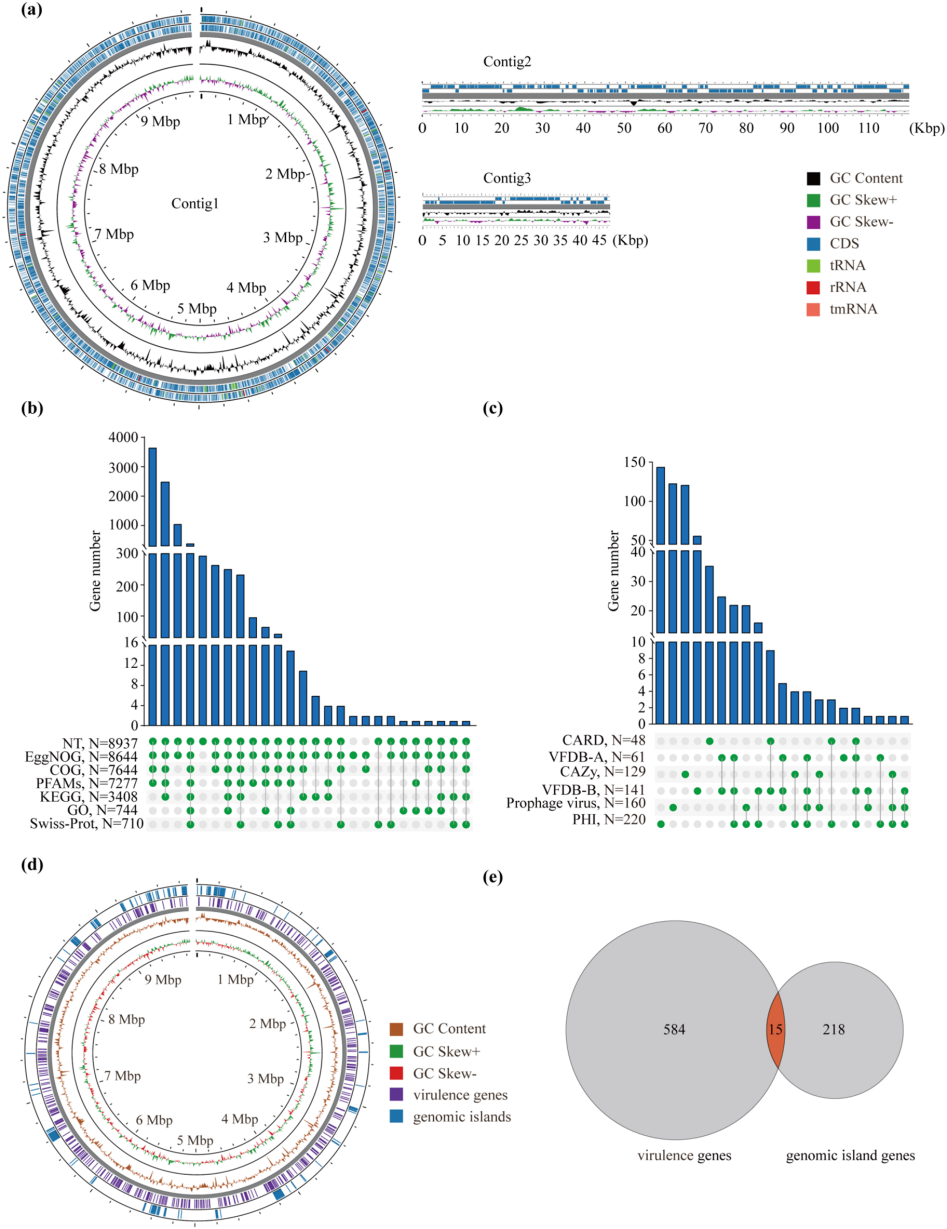
Genome assembly, structural features, and functional annotation of D6. **(a)** Panoramic view of genome assembly: The left panel shows the complete circular genomic map assembled from Contig1, the right panel displays the linear maps of Contig2 and Contig3. The concentric circles and tracks (from inner to outer) represent: genomic coordinates; GC skew (GC Skew+ and GC Skew-); GC content; coding sequences (CDS) on the forward and reverse strands, and the positions of tRNA, rRNA, and tmRNA genes. **(b, c)** General functional gene and virulence gene annotations. The histogram displays the number of shared genes annotated in commonly used general databases **(b)** and specialized virulence/pathogenicity databases **(c)**. Each row represents a database. Green dots connected by gray vertical bars indicate shared genes annotated across different databases, with the number of shared genes shown by the height of the bars above. **(d)** The virulence signature genome map shows the distribution of virulence genes and genomic islands across the entire genome. **(e)** Shared genes between virulence genes and genomic island genes in D6.

The genome assembly of D6 resulted in 3 contigs with a total length of 10,035,877 bp. Specifically, Contig1 was 9,868,305 bp, Contig2 was 119,810 bp and Contig3 was 47,762 bp (**Figure 4a**; **Supplementary Table 4**). The contig N50 reached 9.86 Mb, and the GC content was 70.40% (**Supplementary Table 5**). A total of 9,166 genes were annotated, including 8,991 on Contig1, 44 on Contig2, and 131 on Contig3 (**Supplementary Table 6**). Specifically, 9,061 coding sequences (CDSs), 86 transfer RNAs (tRNAs), 18 ribosomal RNAs (rRNAs), and 1 transfer-messenger RNA (tmRNA) were identified (**Supplementary Table 7**). BUSCO analysis against the *Streptomyces* lineage dataset revealed 99.4% genome completeness based on single-copy orthologs, meeting the high-quality standard (>90%) (**Supplementary Figure 2a**, **Supplementary Table 8**).

Function annotation was performed using 12 databases. In total, 8,937 genes were annotated in the NT database, 8,644 in EggNOG, 7,460 in COG, 7,277 in PFAMs, 3,408 in KEGG, 744 in GO, and 710 in Swiss-Prot (**Figure 4b**; **Supplementary Figure S2b**). Annotation against virulence- and pathogen-related databases identified 220 genes in PHI, 160 in the prophage virus database, 141 in VFDB-B, 129 in CAZy, 61 in VFDB-A and 48 in CARD (**Figure 4c**; **Supplementary Figure 2c**, **Supplementary Table 9**).

A total of 118 GIs were identified in D6 genome, comprising 233 genes with a cumulative length of 1,287,582 bp, which accounts for 12.83% of the whole genome (**Figure 4d**; **Supplementary Table 10**, **S11**). Meanwhile, comprehensive genome-wide analysis of D6 revealed 599 virulence-associated genes, all of which were localized to Contig1; no virulence genes were detected on Contig2 or Contig3 (**Figure 4e**; **Supplementary Table 11**). Notably, 15 of these virulence-associated genes were located on predicted GIs, suggesting potential horizontal gene transfer events (**Figure 4e**). Comparative analysis further identified 556 and 592 virulence-associated genes in LBUM848 and *S.* NRRL2936, respectively (**Supplementary Table 12**).

To determine the taxonomic position of strain D6, phylogenetic analyses were performed using both core genome-wide and single-gene strategies. A maximum-likelihood tree based on the core genomes of 64 *Streptomyces* strains revealed that strain D6 formed a well-supported monophyletic clade together with *S. lincolnensis* strains, indicating that D6 is a member of the *S. lincolnensis* clade (**Supplementary Figure 3**). This phylogenetic relationship was further supported by trees reconstructed from the amino acid sequences of the housekeeping genes *gyrB* and *rpoB*, both of which robustly placed strain D6 within the *S. lincolnensis* clade with high bootstrap support (**Supplementary Figures 4a, b**). The genomic G+C content of strain D6 (70.40%) differed by only 0.61% from that of strain NRRL 2936 (71.01%). Crucially, ANI and dDDH values between strain D6 and strain *S. lincolnensis* NRRL 2936 were 87.2% and 28.9%, respectively, both below the species delineation thresholds (95% and 70%). All these evidence demonstrate that strain D6 is a novel sister species of *S. lincolnensis*.

### 3.5 Comparative genomic collinearity analysis of D6 with related strains

**Figure 5:**
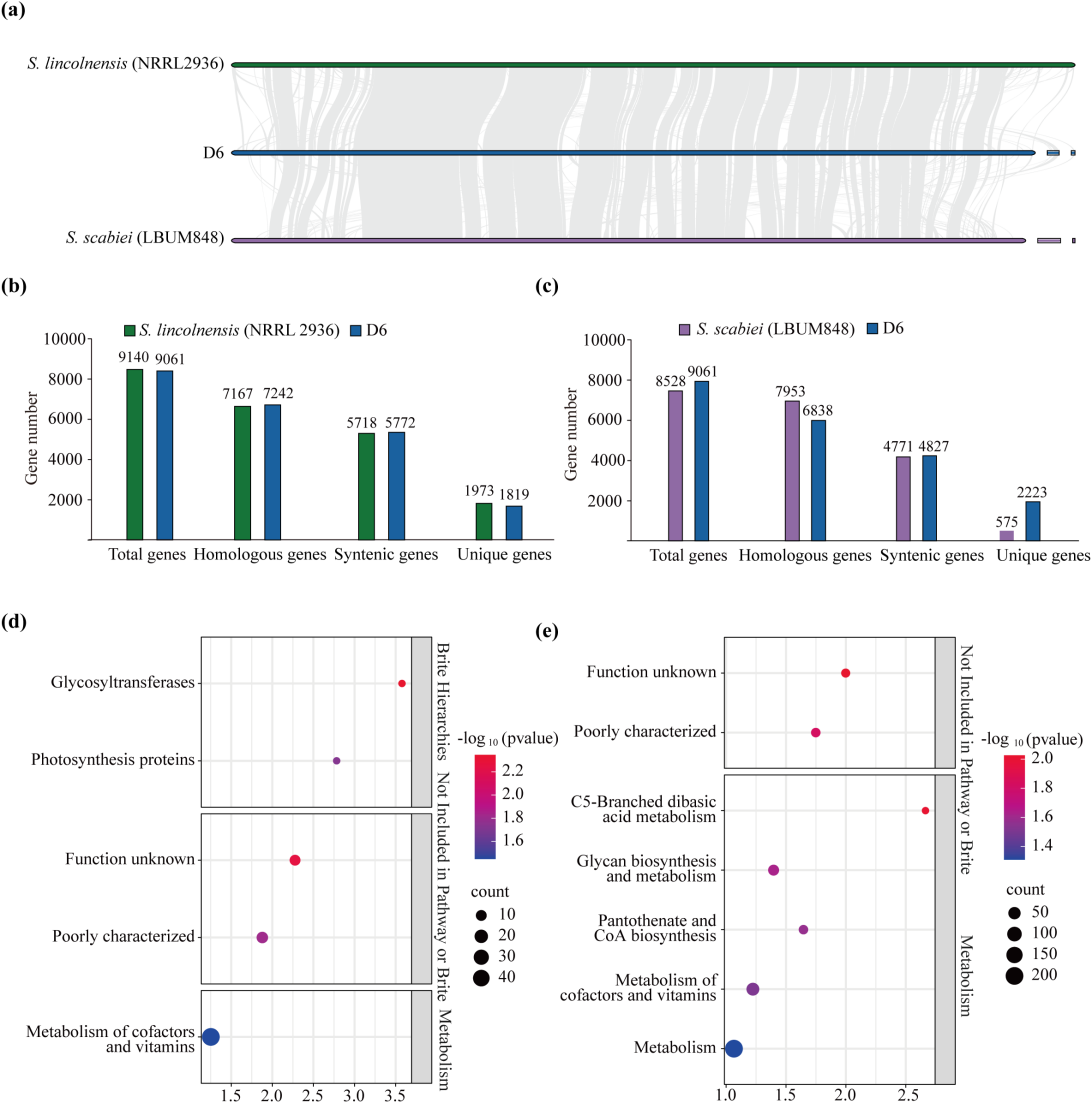
Comparative genomic analysis between D6 and related strains. **(a)** Genomic collinearity analysis. The colinear blocks among the chromosomes of NRRL2936, D6 and LBUM848. **(b, c)** Comparative analyses quantified the number of total genes, homologous genes, collinear genes, and unique genes for each strain in NRRL2936 vs. D6 **(b)** and LBUM848 vs. D6 **(c)**, respectively. **(d, e)** KEGG enrichment analysis of D6-specific genes. The KEGG enrichment analysis compares D6-specific genes against NRRL2936 **(d)** and LBUM848 **(e)**.

Whole-genome collinearity analysis was performed between D6 and NRRL2936 (**Figure 5a**). This analysis identified 11,490 syntenic genes, with 5,718 from D6 and 5,772 from NRRL2936, corresponding to a synteny rate of 63.13% (**Figure 5b**; **Supplementary Table 13**). In addition, 1,819 strain-specific genes were identified in D6, while 1,973 unique genes were detected in NRRL2936 (**Figure 5b**). KEGG enrichment analysis of D6-specific genes revealed significant enrichment for pathways related to glycosyltransferases, photosynthesis proteins, function unknown, poorly characterized and metabolism of cofactors and vitamins (**Figure 5d**). Furthermore, GO enrichment analysis indicated that these genes were significantly enriched in terms associated with S-adenosylmethionine-dependent methyltransferase activity, RNA methyltransferase activity, transferase activity, transferase complexes, macromolecule methylation, RNA methylation, obsolete response to inorganic substance, and methylation (**Supplementary Figure 5a**).

Whole-genome collinearity analysis was also conducted between D6 and LBUM848 (**Figure 5a**). A total of 9,598 syntenic genes were identified, with 4,827 derived from D6 and 4,771 from LBUM848, yielding an overall synteny rate of 54.57% (**Figure 5c**; **Supplementary Table 14**). In addition, 2,223 strain-specific genes were detected in D6, while 575 unique genes were found in LBUM848 (**Figure 5c**). KEGG enrichment analysis of the D6-specific genes revealed significant enrichment for pathways related to function unknown, poorly characterized, C5-branched dibasic acid metabolism, glycan biosynthesis and metabolism, pantothenate and CoA biosynthesis, metabolism of cofactors and vitamins, and metabolism (**Figure 5e**). GO enrichment analysis further indicated significant enrichment in terms associated with external encapsulating structure, cell wall, regulation of molecular function, methylation, macromolecule methylation, RNA methylation, and rRNA methylation (**Supplementary Figure 5b**).

### 3.6 Comparative analysis of virulence genes and identification of key virulence-associated genes in D6

**Figure 6:**
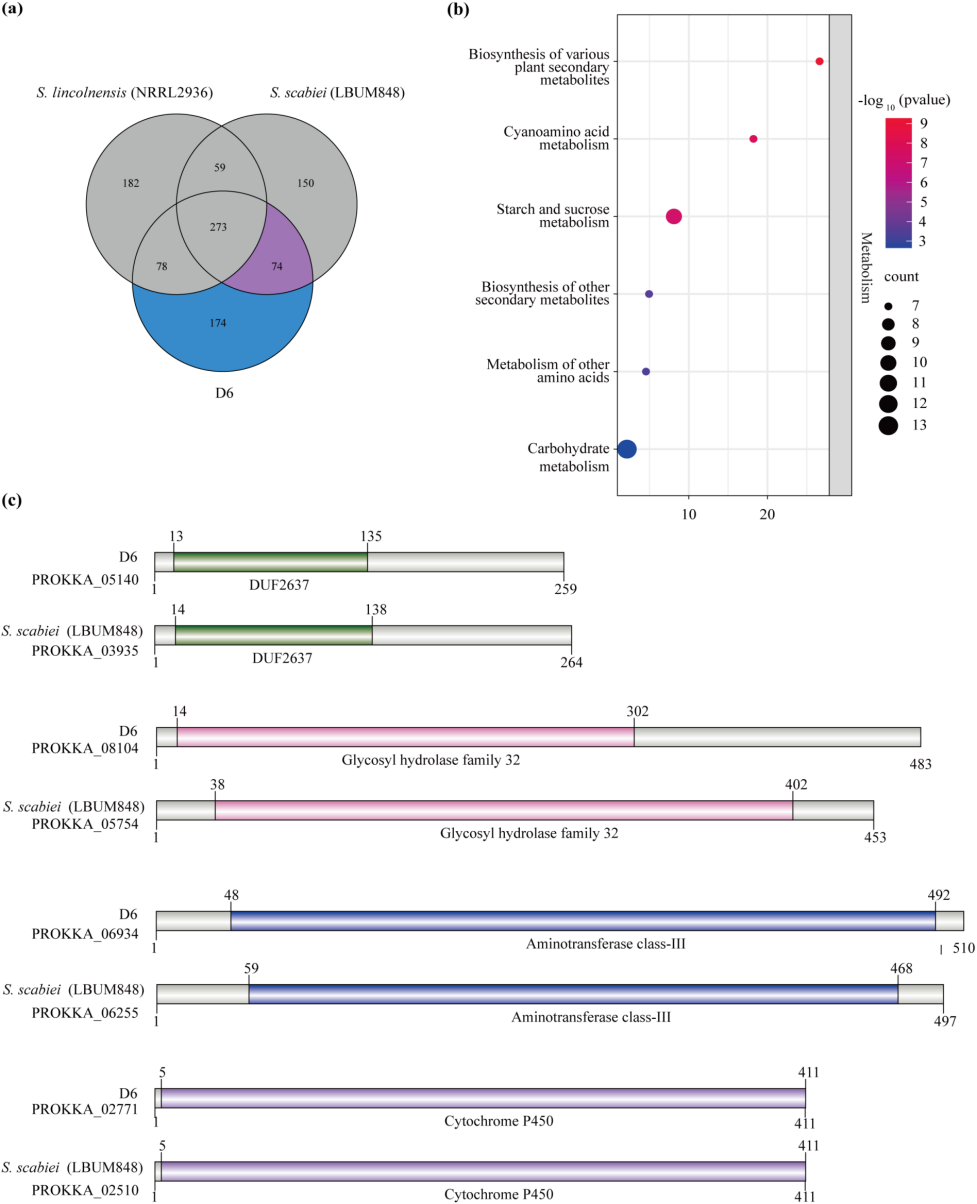
Comparative analysis of virulence factors among D6, NRRL2936, and LBUM848. **(a)** Venn diagram shows the distribution and sharing of virulence-associated genes across the three genomes. The number 74 indicates the core set of virulence genes shared between D6 and the pathogenic model LBUM848. **(b)** KEGG pathway enrichment analysis of the 74 virulence genes shared between D6 and LBUM848. Bar color represents statistical significance [-log10(*P*-value)], with exact values annotated above each bar. **(c)** Domain analysis was performed on the proteins encoded by 4 candidate virulence genes that were screened from the 74 shared genes. Each horizontal bar represents a protein, with colored boxes indicating specific functional domains.

Based on comparative genomic analysis and database annotation, comparative analysis between D6 and NRRL2936 identified 248 putative virulence-associated genes that were present in the D6 genome but absent from the non-pathogenic strain NRRL2936 (**Figure 6a**). To further explore the potential genetic basis underlying the pathogenicity of D6, these candidate genes were compared with those of the pathogenic reference strain *S. scabiei* LBUM848. This analysis revealed a core set of 74 putative virulence-associated genes that are conserved in both pathogenic strains (D6 and LBUM848) but absent in NRRL2936. KEGG enrichment analysis of these 74 shared genes showed significant enrichment in six major metabolic pathways, including biosynthesis of plant secondary metabolites, cyanamino acid metabolism, starch and sucrose metabolism, biosynthesis of other secondary metabolites, metabolism of other amino acids, and carbohydrate metabolism (**Figure 6b**). These pathways have been previously reported to be associated with bacterial adaptation and host-pathogen interactions in plant-associated *Streptomyces*, indicating that the identified genes may be involved in pathogenicity.

Functional annotation of these 74 virulence-associated genes indicated that they can be categorized into two primary groups. The first class includes a substantial number of genes encoding carbohydrate-active enzymes (CAZys), such as members of glycoside hydrolase families *GH3*, *GH20*, *GH32*, and *GH43*, as well as the glycosyltransferase family *GT26*. These enzymes are known to facilitate the degradation of plant cell wall components, thereby contributing to host tissue penetration and colonization. The second class comprises genes with well-established roles in bacterial virulence, including the global transcriptional regulator *Lrp*; key components of the protein homeostasis machinery (*ClpP* and *ClpX*), which are essential for stress tolerance and virulence regulation in diverse bacterial pathogens; nutrient acquisition systems such as the siderophore biosynthesis gene *PvdH* and the enterobactin transport gene *FepC*; and metabolic genes such as *LeuD*, which is involved in leucine biosynthesis (**Supplementary Table 15**).

Genomic localization analysis further revealed that 2 of the 74 virulence-associated genes, *PROKKA_05140* and *PROKKA_08104*, were located on predicted GIs. The former was annotated as a phage-related protein, while the latter encodes a glycoside hydrolase family 32 (GH32) enzyme. The localization of these virulence-associated genes on GIs suggests that horizontal gene transfer (HGT) may have played an important role in the acquisition of virulence traits in D6 (**Supplementary Table 16**).

Based on the 74 virulence-associated genes and well-established role of thaxtomin as the principal virulence determinant in scab-causing *Streptomyces*, 2 potential genes related to thaxtomin biosynthesis were further screened. Among the 2 genes, *PROKKA_06934* encodes a class III pyridoxal-phosphate-dependent aminotransferase, which was classified as a core virulence gene in the VFDB-A (Core Dataset), suggesting its potential involvement in virulence regulation. *PROKKA_02771* encodes a cytochrome P450 enzyme that exhibits strong homology to TxtC (AEL30518.1), a critical enzyme in the thaxtomin biosynthetic pathway (E-value=7.95x10^-32^). This homology indicates the enzyme’s probable role in the biosynthesis of thaxtomin-like secondary metabolites (**Supplementary Table 17**).

To further investigate the structural conservation of the proteins encoded by these 4 candidate genes, domain prediction analysis was conducted on their respective homologs in D6 and the highly virulent reference strain LBUM848. The results confirmed that proteins encoded by *PROKKA_05140*, *PROKKA_08104*, *PROKKA_06934*, and *PROKKA_02771* from D6, as well as their corresponding homologs in LBUM848, harbor the same 4 conserved domains: DUF2637, GH32, class III aminotransferase and cytochrome P450 (**Figure 6c**).

Taken together, these 4 candidate genes, *PROKKA_05140* (phage-related protein), *PROKKA_08104* (GH32), *PROKKA_06934* (class III aminotransferase), and *PROKKA_02771* (cytochrome P450), represent the most plausible virulence determinants in D6. The high degree of domain conservation between D6 and LBUM848 suggests that these genes may perform analogous roles in pathogenicity. Their coexistence indicates that D6 not only retains conserved virulence factors shared among pathogenic *Streptomyces* species but also harbors genetic elements potentially acquired through horizontal gene transfer that may enhance its pathogenic potential. These findings provide robust genomic and structural evidence supporting the previously unrecognized pathogenic potential of the *S. lincolnensis* clade toward potato.

## 4 Discussion

Potato common scab, caused by pathogenic *Streptomyces* species, poses a persistent threat to global potato production by reducing tuber quality and commercial value. Although more than 10 *Streptomyces* species have been associated with this disease (Fyans et al., 2016), the identification of additional pathogenic lineages remains essential for improving our understanding of disease epidemiology and for developing targeted management strategies. In this study, we isolated 31 strains from common scab-infected potato tubers, 9 of which displayed strong inhibitory activity against radish seedlings, and strain D6 with the highest inhibition rate was selected for subsequent analysis. Phylogenomic analysis of core genomes and protein phylogenies of gyrB and rpoB identified D6 as a member of the *S. lincolnensis* clade, and ANI and dDDH analyses confirmed D6 as a distinct sister species of *S. lincolnensis*. This finding identifies D6 as a novel pathogen of potato scab within the *S. lincolnensis* clade, thereby expanding the known pathogenic spectrum of this disease and the taxonomic diversity of plant pathogenic *Streptomyces*.

Pathogenicity assays demonstrated that strain D6 induced severe necrotic lesions on potato tuber slices and exhibited strong inhibitory effects on radish seedling growth (**Figure 1**). Molecular analyses further revealed that D6 harbors key pathogenicity-related genes, including *txtAB* and *tomA* (**Figures 2c**, **S1b, d**). The distinct PAI genotype of D6 (*txtAB*^+^/*nec1*^-^/*tomA*^+^) compared with other characterized strains suggests that pathogenic *Streptomyces s*pecies may employ diverse virulence strategies, highlighting the importance of comprehensive virulence gene profiling to elucidate plant-pathogen interactions.

To elucidate the pathogenic mechanism of D6 from a genomic perspective, we employed PacBio HiFi sequencing to generate the first gapless, chromosome-level genome assembly of this strain. The assembly exhibited high completeness (99.4%) and contiguity (contig N50 = 9.86 Mb), providing a robust resource for functional genomic analyses (**Supplementary Figure 2a**, **Supplementary Table 5**). Genomic annotation identified 599 virulence-associated genes, with 15 localized on GIs. Comparative genomic analysis with the non-pathogenic NRRL2936 and the highly pathogenic LBUM848 uncovered 74 virulence genes conserved in D6 and LBUM848 but absent in the non-pathogenic NRRL2936. Functional enrichment analysis revealed these virulence genes were significantly enriched in pathways related to carbohydrate metabolism, secondary metabolite biosynthesis, and nutrient acquisition, which were critical for plant tissue colonization and pathogenicity (Ma et al., 2024; Li et al., 2024a).

Among the identified 74 virulence genes, 4 genes (*PROKKA_06934*, *PROKKA_02771*, *PROKKA_05140*, and *PROKKA_08104*) were identified based on functional annotation, database classification, and homology to known pathogenic factors (**Figure 6c**; **Supplementary Table 16**, **Supplementary Table 17**). *PROKKA_06934* encodes a class III pyridoxal-phosphate-dependent aminotransferase, which is classified as a core virulence factor in the VFDB-A database. Aminotransferases of this class are known to provide precursors for the synthesis of secondary metabolites, such as phytotoxins and cell wall–modifying compounds (Li et al., 2024b). The same homologous genes in several plant pathogens have been experimentally demonstrated to influence virulence by regulating nitrogen metabolism, redox balance, and toxin biosynthesis pathways (Genin and Denny, 2012; Oh et al., 2017). This suggests a similar role of *PROKKA_06934* in linking the pathogen’s central metabolism to host colonization and pathogenic fitness. *PROKKA_02771* encodes a cytochrome P450 enzyme highly homologous to TxtC, a core enzyme in thaxtomin biosynthesis (Alkhalaf et al., 2019). The presence of the TxtC homolog in pathogenic D6 and LBUM848, but absence in non-pathogenic NRRL2936, suggests that it may mediate toxic secondary metabolite biosynthesis, thereby contributing to pathogenicity and providing compelling genomic evidence for the previously unrecognized pathogenic potential of the *S. lincolnensis* clade. *PROKKA_05140* (encoding a phage-related protein) and *PROKKA_08104* (encoding GH32 enzyme) are both located on GIs, regions of the genome associated with inter-strain variability. The presence of phage-derived genes in virulence-associated regions supports the role of HGT in acquiring pathogenic traits in *Streptomyces* species and highlights their involvement in bacterial adaptation, toxin regulation and secretion system modulation (Fortier and Sekulovic, 2013; Zhang et al., 2016; Chapleau et al., 2016; Tomihama et al., 2016; Francis et al., 2023). Meanwhile, GH32 enzymes hydrolyze sucrose and fructan polymers, potentially disrupting host carbohydrate metabolism and weakening cell wall integrity (Zhang et al., 2017; Bissaro et al., 2015; Holyavka and Artyukhova, 2023). This dual function links plant tissue degradation to metabolic exploitation and highlights their critical auxiliary role in mediating pathogenicity of *D6*. The combined presence of core virulence determinants and these island-associated genes defines a unique genomic signature for D6 that may underlie its specific pathogenicity as a novel *S. lincolnensis* sister species.

In this study, genomic and experimental evidences support that D6 is a novel sister species of *S. lincolnensis* and is capable of causing potato common scab. These findings suggest that the *S. lincolnensis* clade may occupy a broader ecological niche than previously recognized, with members extending from lincomycin production to include plant pathogenicity. The acquisition of virulence genes in D6 may reflect adaptation to agricultural environments with repeated potato host exposure, though the specific selective pressures and evolutionary mechanisms remain to be fully elucidated. These preliminary results provide a basis for future studies on the ecological and evolutionary dynamics of the *S. lincolnensis* clade.

## 5 Conclusion

This study provides the first comprehensive genomic and experimental evidence that strain D6 is a novel sister species of *S. lincolnensis* and a causal agent of potato common scab. Through isolation, pathogenicity assays, and fulfillment of Koch’s postulates, we confirmed the ability of D6 to induce typical scab lesions on potato tubers. The generation of a high-quality, gapless genome (10.04 Mb, 99.4% completeness) using PacBio HiFi sequencing enabled an in-depth analysis of its genomic architecture and virulence determinants. Phylogenomic and phylogenetic analyses identified strain D6 as a member of the *S. lincolnensis* clade, while ANI and dDDH analyses confirmed its taxonomic status as a species distinct from *S. lincolnensis*. Comparative genomic analyses with the non-pathogenic strain *S. lincolnensis* NRRL2936 and the pathogenic reference strain *S. scabiei* LBUM848 identified a core set of 74 putative virulence-associated genes uniquely conserved in the pathogenic strains. These genes were enriched for pathways related to carbohydrate metabolism, secondary metabolite biosynthesis, and nutrient acquisition, all of which represent key functions supporting host colonization and infection. Four candidate virulence genes, *PROKKA_05140* (encoding a phage-derived protein), and *PROKKA_08104* (encoding a GH32 enzyme), *PROKKA_06934* (encoding a class III aminotransferase), *PROKKA_02771* (TxtC homolog, encoding a cytochrome P450), were highlighted as potential pathogenic determinants, reflecting both conserved and possible horizontally acquired mechanisms that may contribute to virulence evolution. Collectively, our findings not only redefine the ecological role of the *S. lincolnensis* clade from an industrial antibiotic producer to a plant pathogen but also provide novel insights into the molecular basis and evolutionary dynamics of virulence in *Streptomyces* species. These results establish a foundation for future functional validation of candidate genes and the development of targeted strategies to mitigate potato common scab.

## Data Availability

The datasets presented in this study can be found in online repositories. The names of the repository/repositories and accession number(s) can be found in the article/**Supplementary Material**.
