# Supplementary material for: Chromosome-level genome assembly and annotation of a novel potato common scab pathogen
Source: Front Plant Sci. 2026 Mar 13;17:1749870. doi: 10.3389/fpls.2026.1749870 (PMC13022933; doi:10.3389/fpls.2026.1749870)
Supplement: Supplementary file 1 [file DataSheet1.docx]

**Supplementary Materials**

**Chromosome-Level Genome Assembly and Annotation of a Novel Potato Common Scab Pathogen**

**Lingling Wei^1#^, Jin Pu^1#^, Hui Du^3^, Rongyan Wang^1^, Xiaowenxuan Gao^1^, Qiangbiao Zhao^4^, Tianjie Wang^4^, Jianli Gao^4^, Decai Yu^2^**^*^**, Guangtao Zhu^1^**^*^**, Jing Liu^1^**^*^

1 Yunnan key Laboratory of Potato Biology, School of Life Sciences, Yunnan Normal University, Southwest United Graduate School, Kunming 650500, China

2 College of Plant Protection,Yunnan Agricultural University, Kunming 650201, China

3 School of Pharmaceutical Sciences, School of Pharmacy and Academy of Chinese Medical Science, Zhejiang Chinese Medical University, Hangzhou, Zhejiang, 310053, China

4 College of Plant Protection, Wenshan Academy of Agricultural Sciences, Wenshan, Yunnan 663000, China

**^#^** These authors contributed equally to this work.

^*^ Correspondence: [13388859299@163.com](mailto:13388859299@163.com) (D. Y.); [zhuguangtao@ynnu.edu.cn](mailto:zhuguangtao@ynnu.edu.cn) (G. Z.); [liujing@ynnu.edu.cn](mailto:liujing@ynnu.edu.cn) (J. L.)


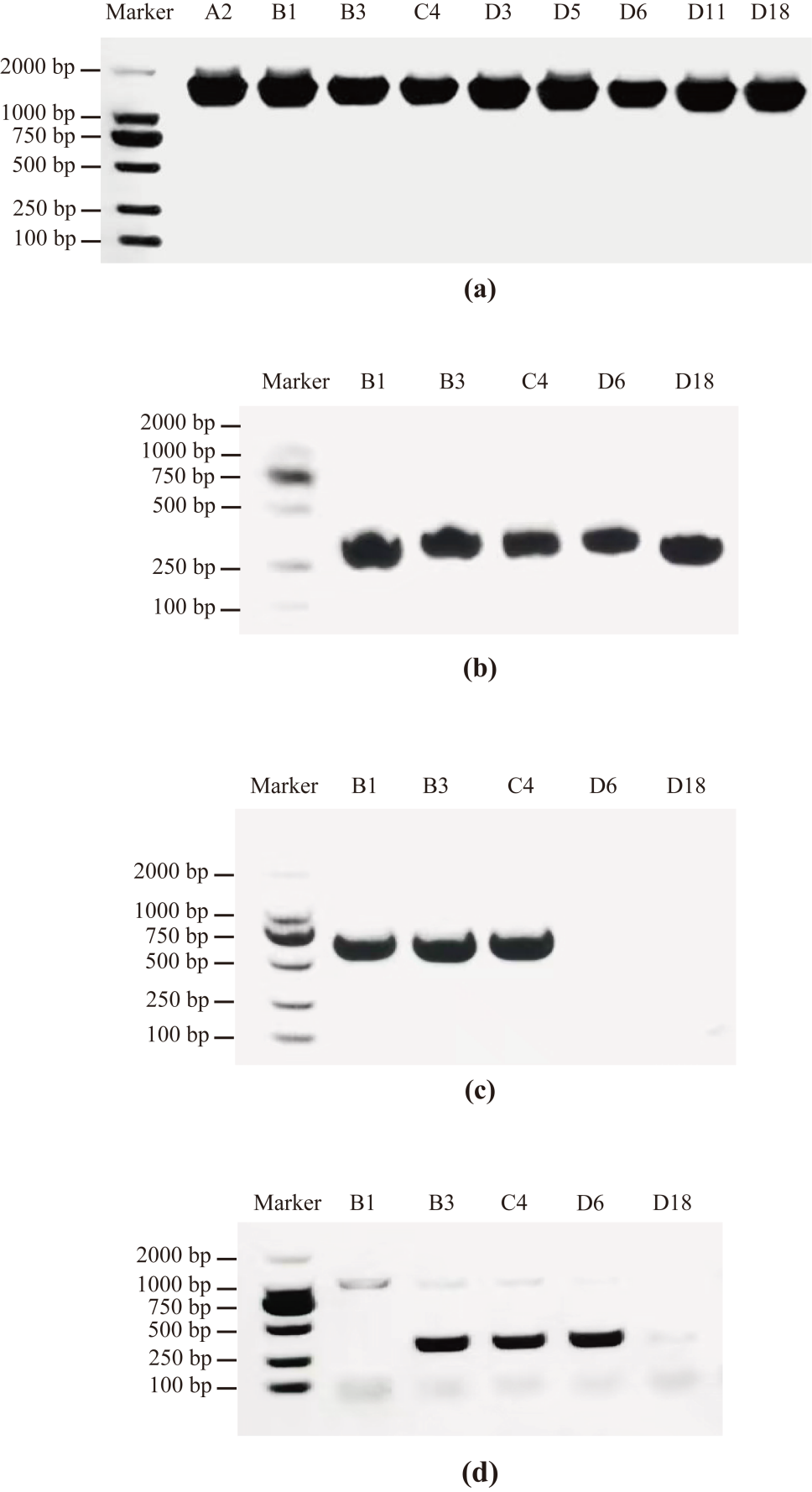


**Figure S1.** 16S rRNA amplification and pathogenic genes detection of the selected strains. (a) 16S rRNA amplification of the 9 strains. (b-d) Pathogenic genes detection. (b) *txtAB*; (c) *nec1*; (d) *tomA*.

**
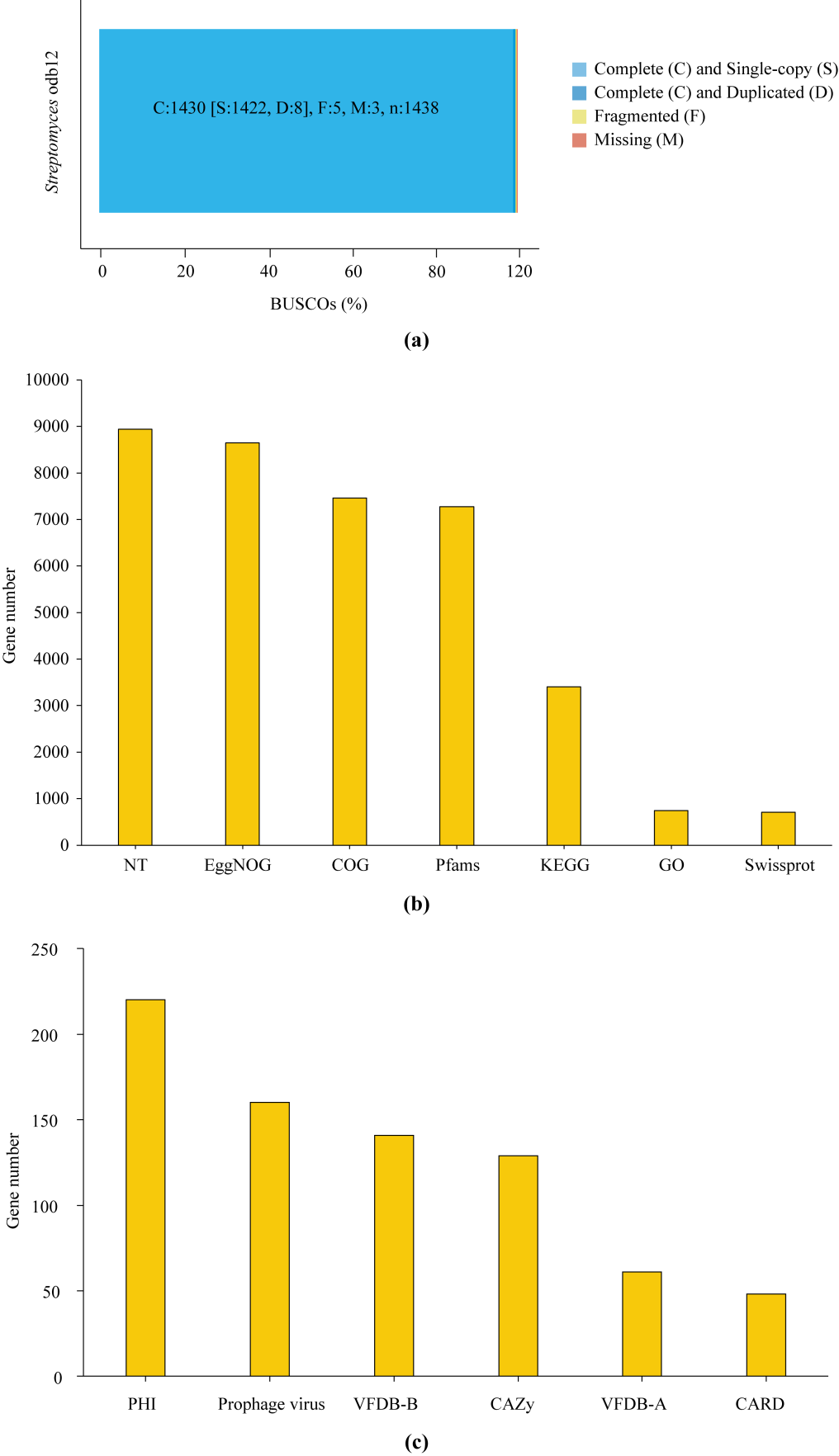
**

**Figure S2.** Genome BUSCO assessment and function annotation. (a) BUSCO evaluation of the D6 genome in the *Streptomyces* database. (b) Function annotation of common gene databases. (c) Functional annotation of virulence-related gene databases.

**
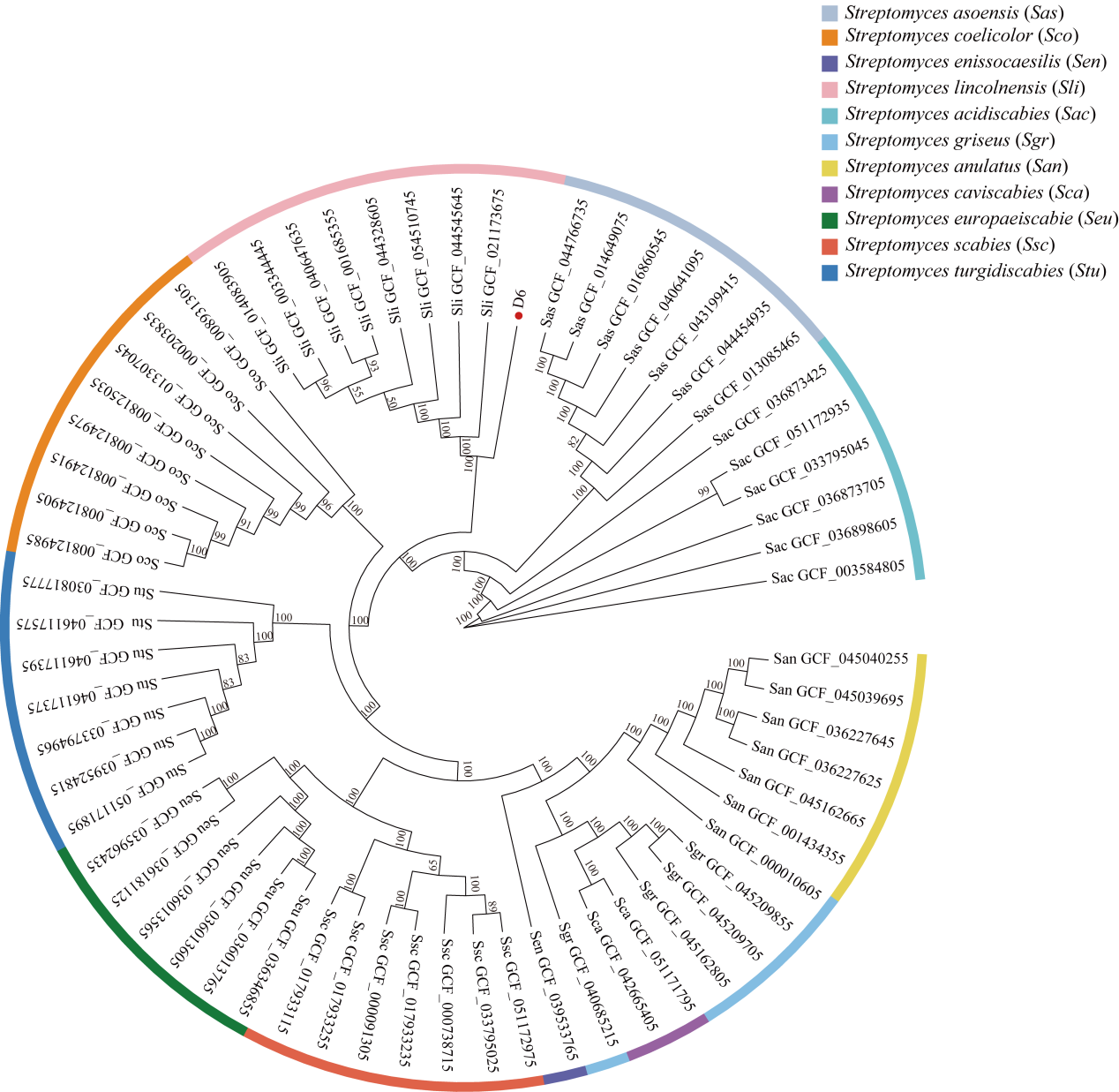
**

**Figure S3.** Whole-genome phylogeny of 64 *Streptomyces* strains. Maximum-likelihood phylogenetic tree based on 1,865 single-copy core genes (591,391 amino acid sites), showing the relationship between strain D6 and 63 reference *Streptomyces* genomes. Strain D6 is highlighted in a red dot. Colors in the outer ring indicate species-level assignments, with strains from the same species depicted in the same color.

**
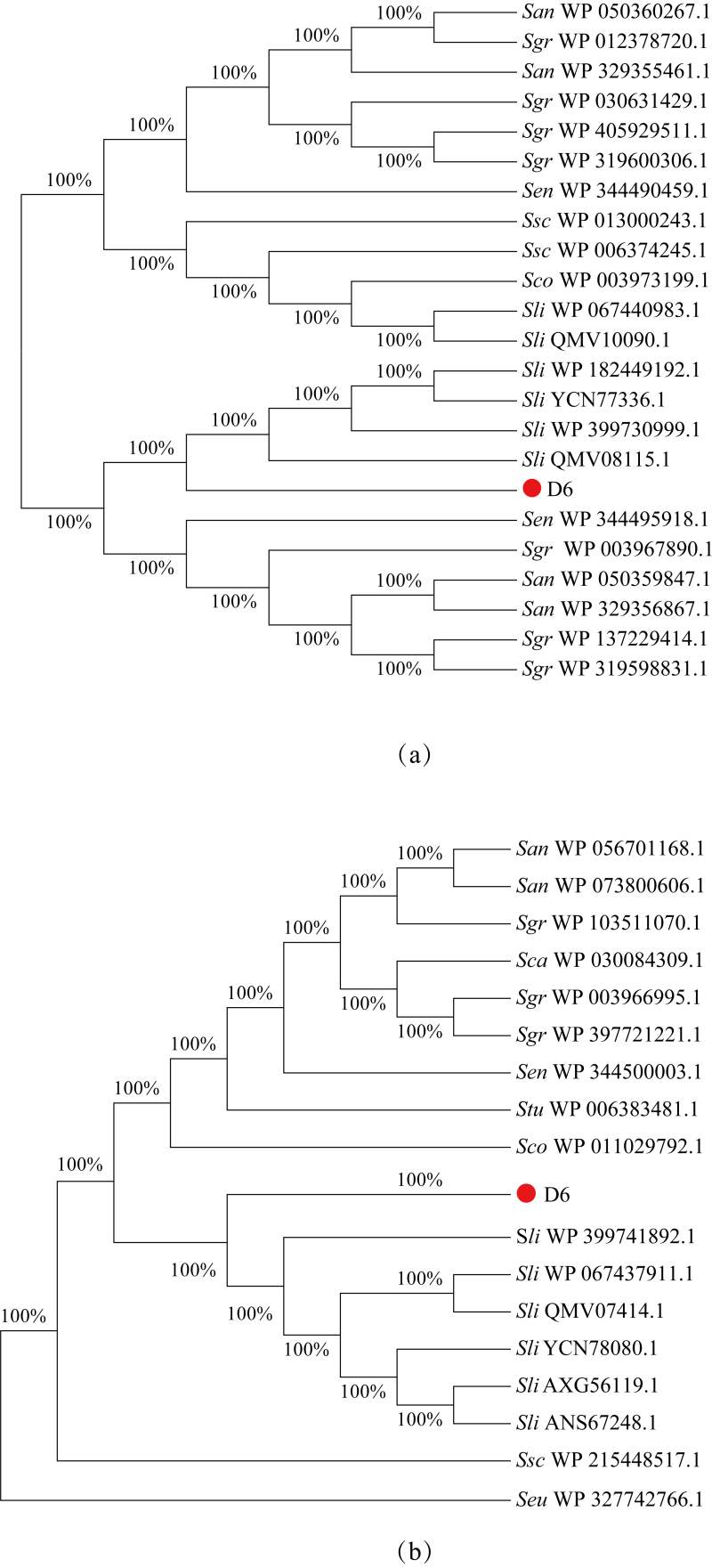
**

**Figure S4.** Protein phylogenetic trees of gyrB and ropB from *Streptomyces* strains. (a) Maximum-likelihood phylogenetic trees reconstructed based on the gyrB (23 strains) protein sequences. (b) Maximum-likelihood phylogenetic trees reconstructed based on the ropB (18 strains) protein sequences.

**
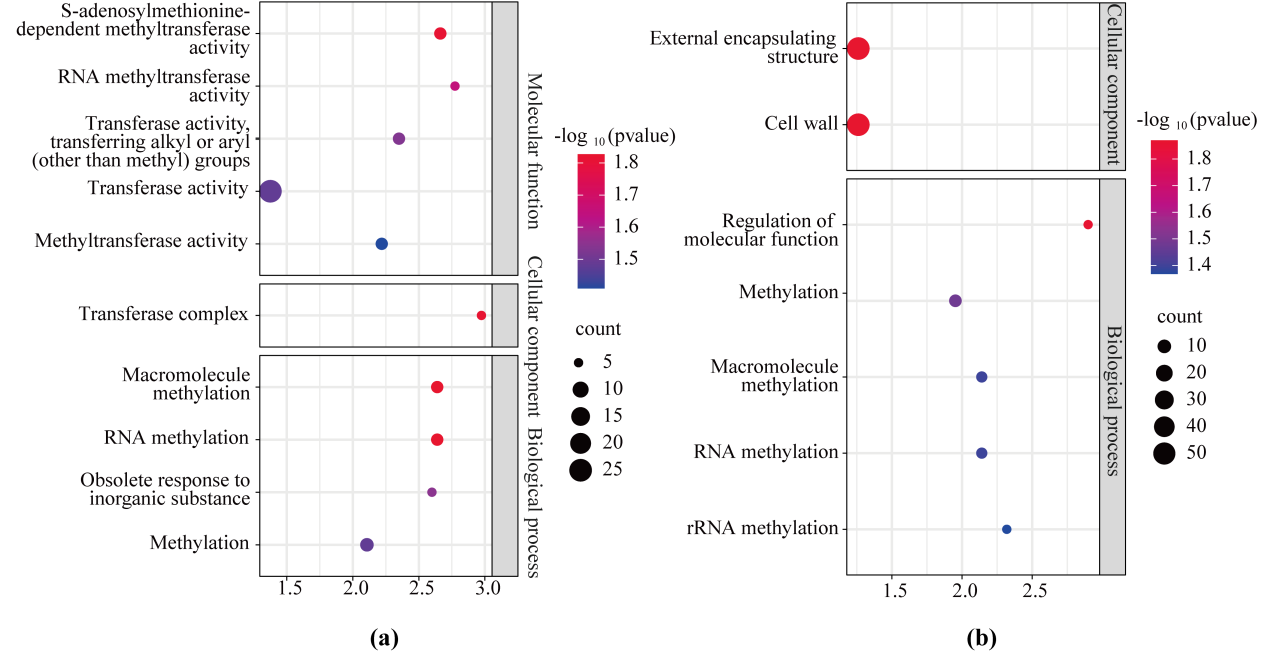
Figure S5.** GO enrichment analysis of D6-specific genes. (a) GO enrichment analysis of D6-specific genes identified via synteny analysis between D6 and *S. lincolnensis* (NRRL2936). (b) GO enrichment analysis of D6-specific genes identified via collinearity analysis between D6 and *S. scabiei* (LBUM848).
